# Predictive Approach to Mapping *Angiostrongylus cantonensis* Nematode Distribution, Canary Islands, Spain

**DOI:** 10.3201/eid3207.251930

**Published:** 2026-07

**Authors:** Lucia Anettová, Jan Divíšek, Radovan Coufal, Anna Šipková, Jana Kačmaříková, Michal Horsák, Vojtech Baláž, Elena Izquierdo-Rodriguez, Barbora Červená, Pilar Foronda, David Modrý

**Affiliations:** Czech University of Life Sciences, Prague, Czech Republic (L. Anettová, D. Modrý); Masaryk University, Brno, Czech Republic (J. Divíšek, R. Coufal, A. Šipková, M. Horsák, D. Modrý); University of Veterinary Sciences, Brno (J. Kačmaříková, V. Baláž, B. Červená); University of La Laguna, Tenerife, Spain (E. Izquierdo-Rodriguez, P. Foronda); Czech Academy of Sciences, Brno (B. Červená); Czech Academy of Sciences, České Budějovice, Czech Republic (D. Modrý)

**Keywords:** Angiostrongylus cantonensis, rat lungworm, parasites, zoonoses, distribution modeling, neuroangiostrongyliasis, eosinophilic meningitis, Tenerife, Canary Islands, Spain

## Abstract

The invasive nematode *Angiostrongylus cantonensis* (rat lungworm) can cause eosinophilic meningitis in humans. Once restricted to Southeast Asia, *A. cantonensis* nematodes are now widespread across the tropics and have been reported in Europe. Tenerife, in the Canary Islands, and the Mediterranean region are emerging hotspots. We surveyed gastropods, rats, and lizards across Tenerife and detected the parasite in all host groups at 2.4%–41.6% prevalence. Using species distribution models, we identified precipitation seasonality as the main driver of habitat suitability; tree cover and climatic variability primarily shaped prevalence patterns. Modeling showed suitable habitats in northeastern Tenerife and several western Canary Islands but limited overlap with areas of dense human population. Multivariate environmental similarity surface analysis comparison with another *A. cantonensis* hotspot, Hawaii, USA, revealed similar environments across the archipelago, except for the novel northeastern Tenerife area. Although no human infections have been reported, continued vigilance is warranted because *A. cantonensis* nematodes are established in Tenerife.

*Angiostrongylus cantonensis* (the rat lungworm) is an invasive parasitic nematode originally endemic to Southeast Asia. Since its original description in China in the 1930s (*1*), the species has exhibited a remarkable capacity for global dispersal. Its range has steadily expanded, and *A. cantonensis* nematodes have been detected on all continents except Antarctica (*2*). Recent records suggest that the parasite is approaching or has already established new foci in or near Europe (*2*–*5*). The rat lungworm is considered a highly successful invader because of its widespread distribution and the invasive potential of its 2 primary host groups: rats as definitive hosts and gastropods as intermediate hosts. Representatives of those host groups are globally invasive and thrive in a wide range of environments (*6*–*9*). The close ecologic association with other successful invaders makes the *A. cantonensis* nematode a striking example of parallel biologic invasion, whereby the parasite expands its range in tandem with the global spread of its hosts.

The rat lungworm is an ecologically successful invader and zoonotic pathogen. In humans, the parasite is the primary cause of eosinophilic meningitis, which is increasingly recognized as a serious infectious disease known as neuroangiostrongyliasis (*10*). Clinical manifestations of *A. cantonensis* infection range from mild influenza-like symptoms to severe neurologic impairment and, in rare cases, death (*10*). In addition to humans, *A. cantonensis* nematodes can infect a wide range of accidental hosts, including other mammals and birds, in which it can cause neurologic symptoms (*11*–*14*).

Despite detection of the parasite in the Mediterranean and Macaronesia, Europe has only had a single reported case of likely autochthonous human neuroangiostrongyliasis, in France (*15*). That single case is in stark contrast to other affected regions, such as Southeast Asia, southern China, and the Pacific Islands, where human cases are relatively common (*16*,*17*). One possible explanation for those differences lies in differences in cultural and dietary practices. In several endemic regions of Asia especially, traditional cuisine includes consumption of raw or undercooked gastropods and paratenic hosts, such as freshwater shrimp, frogs, and monitor lizards, increasing the risk for infection (*18*–*22*), but such practices are rare in Europe. Nevertheless, transmission can occur through accidental ingestion of small, infected gastropods contaminating fresh produce. Infective *A. cantonensis* larvae in gastropod mucus trails has been proposed as a possible route (*23*,*24*), but remains insufficiently demonstrated. Likewise, waterborne *A. cantonensis* transmission has been hypothesized (*25*) but lacks clear supporting evidence. Those possible transmission routes highlight the need for food hygiene and public awareness, even in regions where risky culinary practices are uncommon.

In addition to the newly recognized hotspots within Europe (*3*,*5*,*26*), the Canary Islands, an autonomous community of Spain located in the Atlantic Ocean ≈100 km northwest of Africa, represent a crucial focus of *A. cantonensis* nematodes. Rat lungworm was first reported in Tenerife, Canary Islands, from its definitive hosts in 2010 (*27*) and has been studied extensively in its intermediate and paratenic hosts (*4*,*28*–*30*). By 2025, high *A. cantonensis* prevalences had been reported in host species from the northeastern tip of Tenerife (*28*,*29*), but in the southern part of the island, only 1 positive sample had been documented, from an endemic *Gallotia galloti* lizard (*4*). That apparent spatial disparity might reflect both environmental influences and uneven sampling effort because host collection is generally easier in the humid north.

In contrast to other rat lungworm hotspots with similarly high prevalence in intermediate and paratenic hosts that can serve as sources of human infection (*31*–*33*), no human infection has been reported in the Canary Islands. In comparison, the Pacific Island of Hawaii, USA, another archipelago where *A. cantonensis* nematodes have been established and studied for decades, human infections have been reported since 1959 (*34*). Underdiagnosis cannot be entirely ruled out in the Canary Islands, but this well-developed region has a high standard of healthcare, making underdiagnosis less likely. Although the parasite has almost certainly been in Hawaii longer than in the Canary Islands (*31*), a comparison between climatic conditions could clarify differences in human infections between these 2 invaded archipelagos.

Despite extensive study, the probable distribution and key factors influencing *A. cantonensis* nematode prevalence across the Canary Islands is still unclear, limiting implementation of targeted surveillance. To shed more light on the occurrence of rat lungworm across in the archipelago, and to explain the apparent absence of human infections, we conducted a comprehensive analysis of parasites in 3 different host groups: definitive, intermediate, and paratenic. We also modeled spatial patterns in the distribution and prevalence of *A. cantonensis* nematodes in the Canary Islands and compared climatic conditions between the Canary Islands and Hawaii to assess relative contributions of environmental factors driving those patterns. 

## Materials and Methods

### Sample Collection

**Figure 1:**
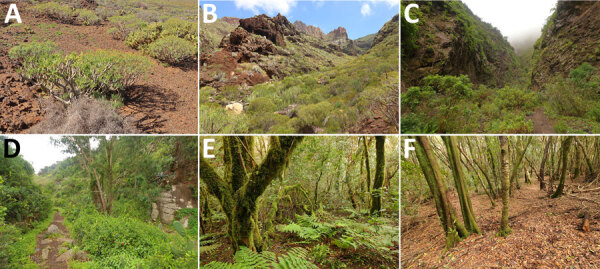
Examples of sampling sites for definitive, intermediate, and paratenic hosts used in a predictive approach to mapping *Angiostrongylus cantonensis* nematode distribution, Canary Islands, Spain. Hosts were collected and sampled in 2022 on Tenerife. A, B) Xeric vegetation types, including bare volcanic rocks with scattered succulents (A) and sparse xerophytic bushland (B). C, D) Bush vegetation, including continuous bush without tree cover (C) and continuous bush interspersed with isolated trees (D). E, F) Laurel forest in Anaga, Tenerife.

**Figure 2:**
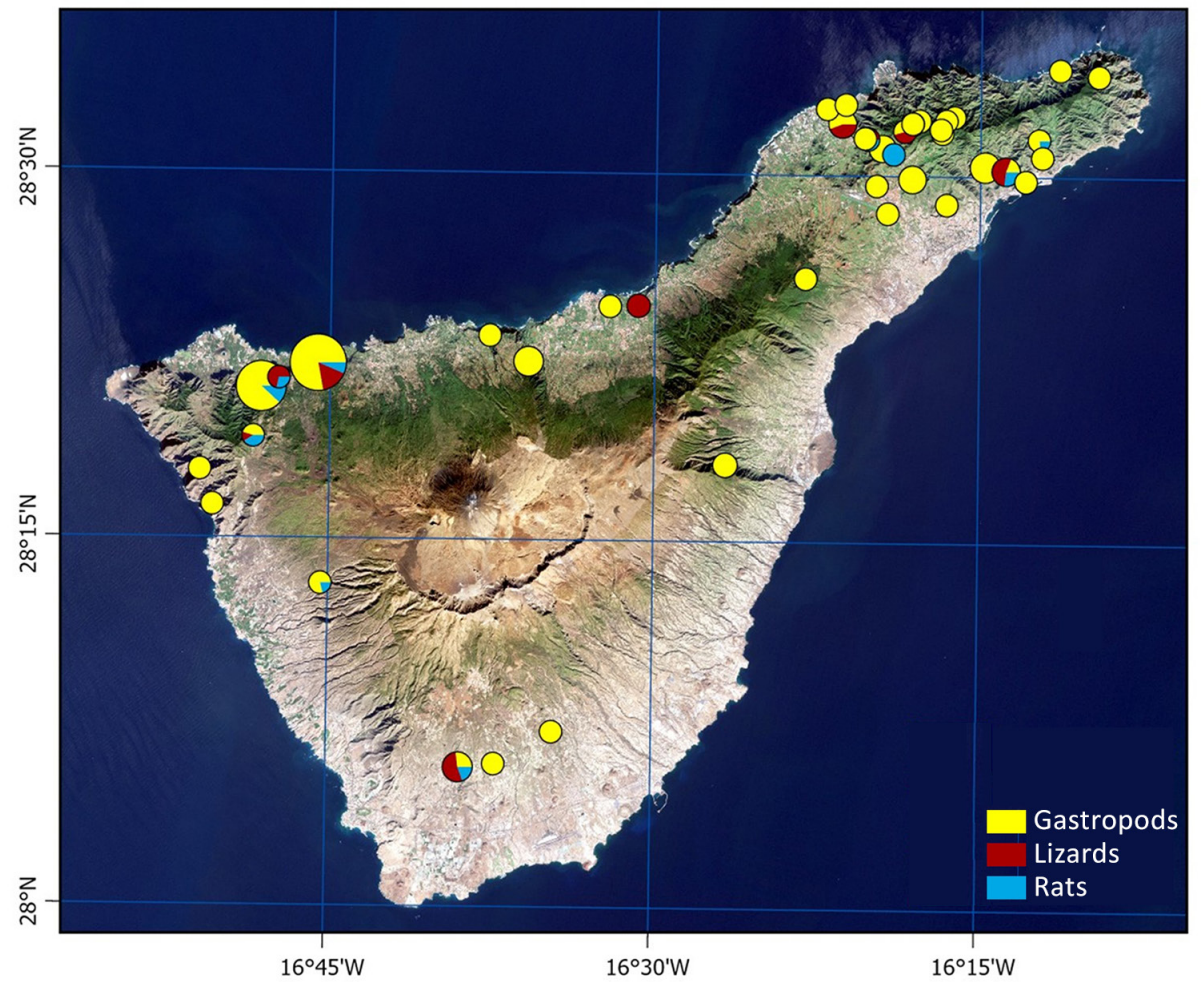
Host sampling sites used in a predictive approach to mapping *Angiostrongylus cantonensis* nematode distribution, Canary Islands, Spain. Host sampling included gastropods intermediate hosts, lizards paratenic hosts, and rats definitive hosts across Tenerife in 2022. We used those samples as the basis for prevalence estimates and species distribution modeling of *A. cantonensis*.

Because Tenerife and the Canary Islands are characterized by diverse landscapes, a wide range of vegetation types, and substantial altitudinal variation (Figure 1), we hypothesized that the presence and prevalence of the nematode would vary considerably across the island. Thus, during April–May 2022, we collected samples from 3 host groups across 41 locations throughout the island, aiming to cover a range of altitude and vegetation zones with varying levels of anthropogenic influence (Figure 2). We considered an area of <400 m^2^ as 1 location. We met all ethical requirements, and animal captures were authorized by the government of the Canary Islands in accordance with applicable law no. 42/2007, under expedient nos. 2022/14555 and 2022/11052. 

#### Rat Collection

We collected definitive rat hosts at 11 locations. We captured rats in live traps set at dusk, left overnight, and retrieved by dawn. A person authorized to handle and euthanize animals euthanized rats by using medetomidine (0.2 mg/kg) and ketamine (3.5 mg/kg), followed by intracardial T61 injection. 

We dissected rats and collected adult nematodes from the heart or pulmonary arteries. We preliminarily identified recovered nematodes as *A. cantonensis* on the basis of macroscopic characteristics and preserved nematodes for subsequent molecular confirmation. We also collected brain samples and stored in 70% ethanol for molecular analysis. We considered rats positive if we confirmed adult nematodes from pulmonary arteries or heart.

#### Lizard Sample Collection

For lizard samples, we used data from our previous study on *A. cantonensis* prevalence in *G. galloti* lizards on Tenerife (*4*). For that study, we collected only tail tissue before releasing lizards. In brief, we captured lizards live in traps baited with a ripe tomato. Traps were checked hourly; we removed captured lizards, grasped the tail to induce caudal autotomy, then released the lizard (*4*). We retained the separated tail, approximately two thirds of the total length, and froze for *A. cantonensis* DNA testing (*4*).

#### Gastropod Collection

We collected gastropods at 37 sites. One person searched each site, an area of ≈20 m^2^ of homogeneous habitat, for ≈60 minutes. We collected both native and nonnative gastropods by handpicking. We did not use the litter sieving method because it primarily captures minute gastropod species that are unlikely to contribute substantially to *A. cantonensis* transmission dynamics. 

We euthanized collected gastropods by freezing and subsequently preserved gastropods in molecular-grade 99% ethanol. We later performed species identification in the laboratory by following the MolluscaBase (https://www.molluscabase.org) nomenclature. Of all collected gastropods, we selected 697 for molecular analysis.

### Molecular Analysis

For molecular analysis, we defrosted lizard tails and weighed, using ≈25 mg of the proximal part of the tail (*4*). For gastropods, we used ≈25 μg of foot tissue; we processed a similar amount of cerebral tissue from the rats. We used the DNeasy Blood & Tissue Kit (QIAGEN, https://www.qiagen.com) to extract DNA from gastropod, lizard, and rat tissues using an extended overnight prelysis step. 

We performed quantitative PCR by using a LightCycler 480 instrument (Roche Life Science, https://lifescience.roche.com), following previously published protocols (*35*). For positive controls, we extracted DNA from a single third-stage *A. cantonensis* larva by the same method used for the samples and diluted ×100. We used nuclease-free water as a negative control. We ran the assay in duplicate and calculated the average cycle threshold (Ct) values between positive sample duplicates by performing absolute quantification analysis of the 2nd derivative maximum. We only considered amplification curves with a Ct value <35 positive to avoid false-positive results caused by amplification and fluorescence artifacts or by cross-contamination.

For adult nematodes, we extracted DNA from approximately the middle third of the body by using innuPREP Forensic Kit (Innuscreen GmbH, https://innuscreen.ist-ag.com). We amplified the complete cytochrome c oxidase subunit I (COI) gene, as previously described (*5*). We separated PCR products and visualized by using agarose gel electrophoresis and subsequently purified by using ExoSAP-IT reagent (Thermo Fisher Scientific, https://www.thermofisher.com). We submitted samples to SEQme (https://www.seqme.eu) for Sanger sequencing. We checked, trimmed and aligned sequences in Geneious Prime 2025.2.2 (https://www.geneious.com) and checked their identity against the GenBank database by using BLASTn (https://blast.ncbi.nlm.nih.gov), according to described methods (*36*).

### Species Distribution Modeling

To model the potential distribution of *A. cantonensis* in the Canary Islands, we used data derived from field surveys. We considered locations positive if we detected the parasite’s DNA by molecular methods in any host species (rats, lizards, or gastropods) or if we found adult nematodes in the pulmonary arteries of rats. 

We performed species distribution modeling by using the maximum entropy algorithm in Maxent software version 3.4.4 (American Museum of Natural History, https://biodiversityinformatics.amnh.org/open_source/maxent). Environmental predictor variables included bioclimatic data, land cover, vegetation indices, and topographic characteristics (Appendix Table 1). We resampled all raster layers to a common resolution of ≈100 m^2^ (0.0012555749) and kept in World Geodetic System 1984 (GIS Geography, https://gisgeography.com/wgs84-world-geodetic-system) to match the coordinate format of occurrence data. To ensure model calibration focused on Tenerife, we used a bias file to constrain background point selection to this island.

We obtained the bioclimatic variables for Tenerife from 1981–2010 from the CHELSA database (https://www.chelsa-climate.org). CHELSA variables included annual mean temperature in degrees Celsius (bio1), temperature seasonality (bio4), annual precipitation in kilograms per square meter (bio12), and precipitation seasonality (bio15) (*37*). We used a 2018 tree cover density (TCD) layer and CORINE land cover (CLC) categories from the European Union’s Copernicus Land Monitoring Service (https://land.copernicus.eu) to represent the landscape structure and transformed TCD and CLC into binary layers. We aggregated CLC classes to define land categories as urban areas (class 1), agricultural land (classes 21, 22, and 24), forests (class 31), scrub vegetation (class 32), pastures and grasslands (classes 231 and 321), and nonvegetated areas (class 33).

We represented local site moisture conditions by a topographic wetness index (TWI), calculated in SAGA GIS version 8.3.0 (https://sagagis.com) on the basis of the Copernicus global and European digital elevation model (Copernicus Data Space Ecosystem, https://dataspace.copernicus.eu) at 90 meters resolution (*38*). We incorporated vegetation dynamics via mean (SD) normalized difference vegetation index (NDVI) for 2022 (*39*). We also included a terrain ruggedness index (TRI) as a topographic predictor. We resampled all raster layers to a common resolution of ≈100 m^2^ (0.0012555749) by using B-spline interpolation in SAGA GIS.

We trained models with default Maxent settings unless stated otherwise. We excluded variables contributing <0.5% to the model to improve model parsimony. We projected the final model to all Canary Islands to identify suitable habitats beyond Tenerife.

We calculated *A. cantonensis* prevalence as the percentage of positive samples for 37 locations in Tenerife. We excluded locations with <6 samples or with samples marked as failed or dubious. To model prevalence, we used a boosted regression tree (BRT) algorithm and the same set of environmental predictors as in the Maxent modeling. We used Mann-Whitney U test to assess differences in prevalence between locations with overlapping host groups and those with a single host group. To assess the overlap between areas with high human population density and predicted suitability for *A. cantonensis* nematodes, we overlaid the Maxent and the prevalence model outputs with land cover data on urban areas from CLC class 1.

We ran the BRT model in R (The R Project for Statistical Computing, https://www.r-project.org) by using the gbm.step function from the dismo package (https://r-packages.io/packages/dismo/circles). We used the following parameters in the BRT model: family = gaussian, tree.complexity = 3, learning.rate = 0.001, bag.fraction = 0.75, and step.size = 10. We selected the optimal number of boosted regression trees via 5-fold cross-validation. We weighted locations by the number of host samples collected per site, ranging from 6 to 106 (mean 23.84) host samples. We ran an initial model with all predictors, then removed predictors with zero contribution before refitting the final model using the same settings.

To assess climatic similarity between the Canary Islands and the Hawaiian Islands, we performed a multivariate environmental similarity surface (MESS) analysis in R by using the dismo and raster packages (https://r-packages.io/packages/dismo/circles). In that analysis, we used bioclimatic variables bio1, bio4, bio12, and bio15, as described. We obtained climatic data for Hawaii and the Canary Islands from the CHELSA database (1981–2010). We trained MESS on climatic data from Hawaii, then projected to the Canary Islands to identify areas of environmental similarity and novelty relative to the training region.

## Results

### *A. cantonensis* Distribution

Of 41 surveyed locations, we confirmed >1 positive host species in 35 locations. In total, we collected 78 rats (both *Rattus rattus* and *R. norvegicus*) from 11 sites; 13 (16.7%) rates tested positive. We observed the highest *A. cantonensis* prevalence in rats in Tegueste, the humid northeast of the island, where half of the examined rats carried the parasite. We confirmed all adult nematodes collected from rats were *A. cantonensis*, and COI sequences were identical across all specimens (GenBank accession no. PX496382). 

Among 129 *G. galloti* lizards examined, 35 (27.1%) were *A. cantonensis* positive (*4*). For lizards, the highest infection rate (63.6%) was in Anaga National Park in the humid northeast. 

Gastropods represented the largest sample set. Among 697 individual gastropods tested from 41 locations, 185 (26.7%) were *A. cantonensis* positive; results for 4 were inconclusive, showing weak or inconsistent amplification signals (Ct values >35) upon repeated quantitative PCR analysis. The Anaga region showed the highest *A. cantonensis* prevalence (43.3%) in gastropods. 

After excluding sites with <6 samples or with failed or dubious results, prevalence estimates calculated for 37 sites ranged from 0 to 0.70 (mean 0.28). Sampling of multiple host groups overlapped in 11 locations, and 30 locations included only 1 host group (Appendix Table 2). Mean prevalence was lower in locations with multiple host groups (17.6%) compared with locations with a single host group (33.7%); however, that difference was not statistically significant (p = 0.15 by Mann-Whitney U test). 

### Predicted Environmental Suitability

**Figure 3:**
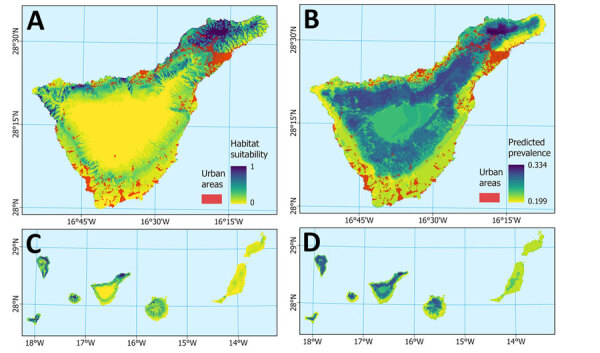
Modeled habitat suitability from a predictive approach to mapping *Angiostrongylus cantonensis* nematode distribution, Canary Islands, Spain, and prevalence of *A. cantonensis* across Tenerife and the Canary Islands based on field survey data (2022). A, C) MaxEnt model predicting habitat suitability (probability of occurrence), with overlap of urban areas (CORINE Land Cover, class 1), for Tenerife detail (A) and the Canary Islands archipelago (C). B, D) Boosted regression tree model predicting prevalence in Tenerife (B) and the Canary Islands archipelago (D).

Maxent predictions showed that the highest habitat suitability is in the northeastern tip of the island, with scattered patches in mid-altitude zones. We retained 8 variables in the final model: precipitation seasonality, annual mean temperature, topographic wetness, terrain ruggedness, TCD, vegetation indices (mean NDVI [SD]), and temperature seasonality. The most influential predictors for *A. cantonensis* detection were precipitation seasonality (52.1% contribution), annual mean temperature (18.1% contribution), and terrain ruggedness (9.5% contribution) (Table 1). Response curves indicated that the parasite favored intermediate temperatures and lower rainfall variability (Appendix Figures 1–3). Intersecting the Maxent prediction with urbanized areas showed limited overlap between highly suitable habitats and densely urbanized zones (i.e., areas of high population density), and overlap mainly is restricted to small patches in the northeastern outcrop (Figure 3).

**Table 1:** Percent contribution and permutation importance of environmental variables retained in a predictive approach to mapping *Angiostrongylus cantonensis* nematode distribution, Canary Islands, Spain*

Variable	% Contribution	Permutation
Precipitation seasonality	52.1	9.6
Mean annual temperature	18.1	49.8
Topographic wetness index	10.4	9.4
Terrain ruggedness index	9.5	20.3
Tree cover density, 2018	8.4	0.9
Mean NDVI (SD), 2022	0.8 (0.5)	1.6 (0.4)
Temperature seasonality	0.4	7.9

*Predictions of habitat suitability (probability of occurrence) were made in MaxEnt mode on the basis of data from *A. cantonensis* field surveys conducted on Tenerife in 2022. We used bioclimatic variables from the CHELSA database (https://www.chelsa-climate.org) for 1981–2010 (*37*), including annual mean temperature in degrees Celsius (bio1), temperature seasonality (bio4), annual precipitation in kilograms per square meter (bio12), and precipitation seasonality (bio15). NDVI, normalized difference vegetation index.

### Influences on Parasite Prevalence

The BRT model (pseudo-R^2^ = 26%, mean squared error = 0.0412) confirmed that TCD (30.9%), precipitation seasonality (22.2%), and temperature seasonality (17.0%) were the strongest predictors of prevalence variation among sites. Vegetation indices also contributed notably (NDVI mean 12.7% [SD 6.9%]). In contrast, annual precipitation and topographic wetness had minimal influence (1%). The BRT model predicted prevalence values ranged from 20% to 32% across Tenerife. Predicted prevalence maps highlighted foci overlapping with the northeast but also indicated suitable mid-elevation habitats outside the current known *A. cantonensis* distribution (Table 2, Figure 3).

**Table 2:** Relative influence of environmental variables in boosted regression tree model used in a predictive approach to mapping *Angiostrongylus cantonensis* nematode distribution, Canary Islands, Spain*

Variable	% Relative influence
Tree cover density	30.95
Precipitation seasonality	22.19
Temperature seasonality	17.03
Mean NDVI (SD)	12.74 (6.88)
Mean annual temperature	5.83
Terrain ruggedness index	3.21
Annual precipitation	0.79
Topographic wetness index	0.36

*Model used local prevalence of *A. cantonensis* nematode detection among multiple host groups across Tenerife on the basis of 2022 field surveillance data. NDVI, normalized difference vegetation index.

### Climatic Conditions Associated with *A. cantonensis* Distribution

**Figure 4:**
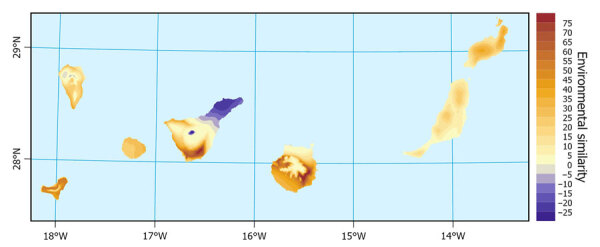
Multivariate Environmental Similarity Surface (MESS) analysis assessing climatic similarity between Hawaii, USA (training region with known human cases of neuroangiostrongyliasis), and the Canary Islands using selected bioclimatic variables in a predictive approach to mapping *Angiostrongylus cantonensis* nematode distribution, Canary Islands, Spain. Values indicate areas of environmental similarity and novelty relative to Hawaii.

MESS projections using bioclimatic variables bio1, bio4, bio12, and bio15 indicated relatively high environmental similarity to Hawaii in the drier leeward areas of southern Tenerife, southern Gran Canaria, and El Hierro, but the humid northeast of Tenerife showed low similarity and had novel conditions relative to Hawaii (Figure 4). Those projections suggest that climatic analogy to conditions in Hawaii occur primarily in drier Canary Island habitats, whereas the humid northeastern sector represents a distinct environmental space that might follow its own transmission pathways.

## Discussion

Our survey confirms that *A. cantonensis* is now firmly established across Tenerife. The parasite was detected across the island, and 35 of 41 surveyed locations tested positive, including arid southern sites where the parasite was previously reported in endemic lizards (*4*). 

The invasion process is typically described as a sequence of 5 stages: transport, introduction, establishment, spread, and negative impacts (*40*). Establishment can be recognized by the successful exploitation of local hosts and an increasing number of infected host species over time (*41*,*42*). Since the parasite was first recorded on Tenerife in 2010 (*27*), it circulated among both endemic and invasive species, involving intermediate as well as paratenic hosts (*28*,*30*). Therefore, we consider *A. cantonensis* nematodes to be well established on Tenerife. Distribution on the remaining Canary Islands is less clear, but the parasite has been documented in intermediate and accidental hosts on other islands in the archipelago (*43*). Involvement of multiple host taxa, including numerous gastropods species, lizards, and rats (Appendix Table 2), raises concerns not only for zoonotic risk but also for potential impacts on wildlife that could suffer from neurologic disease caused by *A. cantonensis* infection.

Although locations with multiple host groups might be expected to enable completion of the parasite life cycle, we did not observe higher *A. cantonensis* prevalence in those sites. That observation might reflect differences in sampling effort, host-specific infection dynamics, or environmental conditions rather than true transmission intensity. Thus, the presence of multiple host types alone might not be a sufficient indicator of elevated transmission risk.

Despite the well-documented presence of *A. cantonensis* nematodes in animal hosts, no human neuroangiostrongyliasis cases have been reported from Tenerife, mirroring a pattern from the Mediterranean where the parasite has been confirmed but no cases of human infection have been reported (*3*,*5*). One plausible explanation lies in local cultural and culinary practices. In contrast to Southeast Asia and parts of the Pacific, where raw or undercooked gastropods (including small or hidden gastropods on vegetation or produce), freshwater shrimp, frogs, or lizards are often consumed (*18*–*21*), such practices are not common in Europe. Sporadic cases in the future cannot be entirely ruled out, and although dietary habits and food preparation standards likely reduce infection risk, accidental exposure via produce contaminated with small gastropods remains possible.

In Tenerife specifically, our models highlight another potential factor: limited overlap between areas of highest predicted environmental suitability for *A. cantonensis* nematodes and densely populated urban zones. That overlap was restricted to small patches in the humid northeast. Instead, most of the suitable habitat is associated with forested or seminatural areas, where human–gastropod contact is expected to be less frequent. The parasite’s dependence on precipitation seasonality and TCD, as identified by Maxent and BRT analyses, reinforces that pattern because those conditions are most pronounced in natural and forested landscapes rather than in croplands or densely urbanized areas.

Of note, the BRT model explained only 26% of variation in prevalence values and produced predictions in a narrow range of 20%–32%, rather than across the full 0%–70% prevalence spectrum. Although the model was unable to predict exceptionally high prevalences, the spatial pattern was well captured, confirming the results of Maxent modeling.

The MESS analysis revealed variable climatic similarity across the Canary Islands. Drier leeward zones climatically resemble Hawaii, but the humid northeastern tip of Tenerife, where *A. cantonensis* prevalence is highest, falls outside the Hawaii reference climate. That apparent paradox illustrates that bioclimatic factors alone cannot predict parasite presence or human infection risk. Thus, distinction is needed between parasite establishment in natural hosts, shaped by climate and rat ecology, and human case emergence, which depends on ecologic, cultural, and socioeconomic exposure drivers.

Comparisons with other regions support that view. In Hawaii, where the parasite was introduced and is now widespread, cases of human infection have been reported (*31*), possibly related to anthropologic and ecologic factors, including fresh produce carrying small infected gastropods or, potentially, their mucus. On the other hand, in Australia, neuroangiostrongyliasis cases are more commonly reported in dogs (*44*), again reflecting local exposure pathways rather than climate alone. Those examples suggest that the parasite’s broad ecologic tolerance and ability to persist in rats and snails effectively decouple its distribution from strict climatic boundaries. *A. cantonensis* nematodes have the capacity to overwinter in rodents because of long patent periods and have been experimentally shown to persist in snails (*45*), further widening the climatic niche beyond those captured by environmental predictors.

Limitations of this study include nonuniform host sampling across locations, single time-point prevalence estimates, and a MESS analysis restricted to climatic similarity without accounting for ecologic differences. Nonetheless, integrating field data with modeling offers a practical framework for identifying areas of potential risk.

In conclusion, our results highlight that species distribution models are useful for identifying environmental hotspots for *A. cantonensis* nematodes and guiding surveillance but cannot predict human infection risk alone. Future research should integrate ecologic modeling with epidemiologic and anthropologic perspectives, addressing dietary practices, food safety, water systems, and human behaviors that modulate zoonotic potential. The absence of human cases despite locally established host cycles in Tenerife, Mallorca, Valencia, and Naples suggests exposure-related factors are critical determinants. Nonetheless, continued vigilance for human *A. cantonensis* infection is warranted in areas with established host populations.

Appendix Additional information on predictive approach to mapping *Angiostrongylus cantonensis* nematode distribution, Canary Islands, Spain.
