# Supplementary material for: Predictive Approach to Mapping Angiostrongylus cantonensis Nematode Distribution, Canary Islands, Spain
Source: Emerg Infect Dis. 2026 Jul;32(7):1113–22. doi: 10.3201/eid3207.251930 (PMC13322411; doi:10.3201/eid3207.251930)
Supplement: Appendix — Additional information on predictive approach to mapping Angiostrongylus cantonensis nematode distribution, Canary Islands, Spain. [file 25-1930-Techapp-s1.pdf]

Article DOI: https://doi.org/10.3201/eid3207.251930

EID cannot ensure accessibility for supplementary materials supplied by authors. Readers who have difficulty accessing supplementary content should contact the authors for assistance.

Predictive Approach to Mapping *Angiostrongylus cantonensis* Nematode Distribution, Canary Islands, Spain

Appendix

Appendix Table 1. Environmental predictor variables used in MaxEnt and Boosted Regression Tree (BRT) models to assess habitat suitability and prevalence of *Angiostrongylus cantonensis* across Tenerife, based on field survey data. All raster layers were resampled to ≈100 m resolution and projected in the WGS 1984 coordinate system.

Variable	Description	Source
bio1	Annual Mean Temperature (1981–2010)	https://chelsa-climate.org/
bio4	Temperature Seasonality (1981–2010)	https://chelsa-climate.org/
bio12	Annual Precipitation (1981–2010)	https://chelsa-climate.org/
bio15	Precipitation Seasonality (1981–2010)	https://chelsa-climate.org/
TCD_2018_100m_eu_03035_V2_0	Tree Cover Density (%)	https://land.copernicus.eu/en/products/high-resolution-layer-tree-cover-density
Agricultural_land_CLC21–22–24	CORINE Land Cover classes 2.1 + 2.2 + 2.4	https://land.copernicus.eu/en/products/corine-land-cover
Forests_CLC31	CORINE Land Cover class 3.1	https://land.copernicus.eu/en/products/corine-land-cover
Non-vegetated_areas_CLC33	CORINE Land Cover class 3.3	https://land.copernicus.eu/en/products/corine-land-cover
Pastures-grasslands_CLC231–321	CORINE Land Cover classes 231 + 321	https://land.copernicus.eu/en/products/corine-land-cover
Scrub_vegetation_CLC32	CORINE Land Cover class 3.2	https://land.copernicus.eu/en/products/corine-land-cover
Urban_areas_CLC1	CORINE Land Cover class 1	https://land.copernicus.eu/en/products/corine-land-cover
SAGA_TWI	Topographic Wetness Index calculated in SAGA GIS based on Copernicus Global DEM (90 m resolution)	European Space Agency (2024). Copernicus Global Digital Elevation Model. https://doi.org/10.5069/G9028PQB
NDVI_Mean	Normalized Difference Vegetation Index, mean for 2022	https://land.copernicus.eu/en/products/vegetation/normalised-difference-vegetation-index-v2-0-300m
NDVI_SD	Annual variability (2022) expressed as standard deviation of NDVI values	https://land.copernicus.eu/en/products/vegetation/normalised-difference-vegetation-index-v2-0-300m
TRI	Terrain Ruggedness Index (derived from Copernicus Global DEM)	European Space Agency (2024). Copernicus Global Digital Elevation Model. https://doi.org/10.5069/G9028PQB

Appendix Table 2. Gastropod and vertebrate host species sampled across locations in Tenerife and their prevalence of *A. cantonensis* infection, based on field survey data from multiple host groups.

Locality	Coordinates	Species	Group	No.	% Prevalence
Anaga	28.536530, –16.302540	*Rattus rattus* (Linnaeus, 1758)	rats	9	22.2
Anaga_Cuadras_don_Benito	28.536424, −16.302746	*Hemicycla bidentalis* (Lamarck, 1822)	gastropods	8	62.5
Anaga_Mirador_Escobon	28.538271, −16.297338	*Hemicycla bidentalis* (Lamarck, 1822)	gastropods	13	46.2
Anaga_Mirador_Escobon	28.538271, −16.297338	*Insulivitrina lamarckii* (A. Férussac, 1821)	gastropods	10	90.0
Anaga_Pedro_ravine	28.531214, −16.308671	*Gallotia galloti* (Oudart, 1839)	lizards	11	63.6
Anaga_Pedro_ravine	28.531214, −16.308671	*Insulivitrina lamarckii* (A. Férussac, 1821)	gastropods	13	46.2
Anaga_Taborno	28.540924, −16.270456	*Hemicycla bidentalis* (Lamarck, 1822)	gastropods	4	0.0
Anaga_Taborno	28.540924, −16.270456	*Insulivitrina lamarckii* (A. Férussac, 1821)	gastropods	18	11.1
Bajamar	28.548939, −16.354348	*Otala lactea* (O. F. Müller, 1774)	gastropods	3	0.0
Barranco_de_Badajoz_Guimar	28.302850, −16.444870	*Ambigolimax valentianus* (A. Férussac, 1821)	gastropods	7	0.0
Barranco_de_Badajoz_Guimar	28.302850, −16.444870	*Ferussacia folliculum* (Schröter, 1784)	gastropods	20	55.0
Benijo	28.573281, −16.188972	*Pseudosuccinea columella* (Say, 1817)	gastropods	10	40.0
Casa_del_Rio_Anaga	28.538479, −16.276595	*Insulivitrina lamarckii* (A. Férussac, 1821)	gastropods	17	58.8
Cruz_del_Carmen	28.529943, −16.279738	*Hemicycla bidentalis* (Lamarck, 1822)	gastropods	1	100.0
Cruz_del_Carmen	28.529943, −16.279738	*Insulivitrina lamarckii* (A. Férussac, 1821)	gastropods	20	35.0
Cueva_Bermeja	28.4973967, 16.2150136	*Ferussacia folliculum* (Schröter, 1784)	gastropods	3	0.0
El_Rincon	28.410708, −16.512897	*Gallotia galloti* (Oudart, 1839)	lizards	24	45.8
Erjos	28.319297, −16.808729	*Ambigolimax valentianus* (A. Férussac, 1821)	gastropods	3	0.0
Erjos	28.319297, −16.808729	*Deroceras invadens* Reise, Hutchinson, Schunack & Schlitt, 2011	gastropods	5	0.0
Erjos	28.319297, −16.808729	*Gallotia galloti* (Oudart, 1839)	lizards	3	0.0
Erjos	28.319297, −16.808729	*Insulivitrina lamarckii* (A. Férussac, 1821)	gastropods	2	0.0
Erjos	28.319297, −16.808729	*Rattus rattus* (Linnaeus, 1758)	rats	9	0.0
Garachico	28.369344, −16.759317	*Ambigolimax valentianus* (A. Férussac, 1821)	gastropods	19	10.5
Garachico	28.369344, −16.759317	*Deroceras* cf. *reticulatum* (O. F. Müller, 1774)	gastropods	14	0.0
Garachico	28.369344, −16.759317	*Gallotia galloti* (Oudart, 1839)	lizards	17	29.4
Garachico	28.369344, −16.759317	*Insulivitrina lamarckii* (A. Férussac, 1821)	gastropods	30	50.0
Garachico	28.369344, −16.759317	*Physella acuta* (Draparnaud, 1805)	gastropods	10	0.0
Garachico	28.369344, −16.759317	*Pseudosuccinea columella* (Say, 1817)	gastropods	11	0.0
Garachico	28.369344, −16.759317	*Rattus rattus* (Linnaeus, 1758)	rats	7	28.6
Grenadilla_de_Abona	28.120077, −16.576603	*Ambigolimax valentianus* (A. Férussac, 1821)	gastropods	1	100.0
Grenadilla_de_Abona	28.120077, −16.576603	*Otala lactea* (O. F. Müller, 1774)	gastropods	8	25.0
Chamorga	28.569102, −16.158997	*Ambigolimax valentianus* (A. Férussac, 1821)	gastropods	1	0.0
Chamorga	28.569102, −16.158997	*Deroceras* cf. *reticulatum* (O. F. Müller, 1774)	gastropods	3	0.0
Chamorga	28.569102, −16.158997	*Hemicycla bidentalis* (Lamarck, 1822)	gastropods	1	0.0
Chamorga	28.569102, −16.158997	*Insulivitrina lamarckii* (A. Férussac, 1821)	gastropods	2	50.0
Chamorga	28.569102, −16.158997	*Rumina decollata* (Linnaeus, 1758)	gastropods	5	0.0
Chirche	28.219702, −16.755661	*Ambigolimax valentianus* (A. Férussac, 1821)	gastropods	8	0.0

Locality	Coordinates	Species	Group	No.	% Prevalence
Chirche	28.219702, −16.755661	*Deroceras reticulatum* (O. F. Müller, 1774)	gastropods	6	0.0
Chirche	28.219702, −16.755661	*Insulivitrina lamarckii* (A. Férussac, 1821)	gastropods	1	0.0
Chirche	28.219702, −16.755661	*Rattus rattus* (Linnaeus, 1758)	rats	4	0.0
Jover	28.545915, −16.368692	*Planorbella duryi* (Wetherby, 1879)	gastropods	10	10.0
La_Esperanza	28.430139, −16.384239	*Insulivitrina lamarckii* (A. Férussac, 1821)	gastropods	5	100.0
La_Laguna_Baldios	28.475006, −16.321483	*Deroceras reticulatum* (O. F. Müller, 1774)	gastropods	12	58.3
La_Laguna_Baldios	28.475006, −16.321483	*Ferussacia folliculum* (Schröter, 1784)	gastropods	4	75.0
La_Vega_Lagunera_La_Laguna	28.498496, −16.302674	*Deroceras reticulatum* (O. F. Müller, 1774)	gastropods	4	50.0
La_Vega_Lagunera_La_Laguna	28.498496, −16.302674	*Rumina decollata* (Linnaeus, 1758)	gastropods	12	58.3
La_Vega_Lagunera_La_Laguna	28.498496, −16.302674	*Theba pisana* (O. F. Müller, 1774)	gastropods	19	57.9
La_Vera	28.389483, −16.627065	*Ambigolimax valentianus* (A. Férussac, 1821)	gastropods	2	50.0
Las_Cuevas	28.507056, −16.246499	*Ambigolimax valentianus* (A. Férussac, 1821)	gastropods	3	0.0
Las_Cuevas	28.507056, −16.246499	*Deroceras invadens* Reise, Hutchinson, Schunack & Schlitt, 2011	gastropods	4	0.0
Las_Cuevas	28.507056, −16.246499	*Ferussacia folliculum* (Schröter, 1784)	gastropods	35	0.0
Las_Gavias_La_Laguna	28.493779, −16.330084	*Ambigolimax valentianus* (A. Férussac, 1821)	gastropods	2	100.0
Las_Gavias_La_Laguna	28.493779, −16.330084	*Ferussacia folliculum* (Schröter, 1784)	gastropods	2	0.0
Las_Gavias_La_Laguna	28.493779, −16.330084	*Rumina decollata* (Linnaeus, 1758)	gastropods	2	0.0
Las_Gavias_La_Laguna	28.493779, −16.330084	*Theba pisana* (O. F. Müller, 1774)	gastropods	14	50.0
Las_Mercedes	28.532432, −16.280770	*Insulivitrina lamarckii* (A. Férussac, 1821)	gastropods	8	25.0
Maria_Jimenez	28.504414, −16.230469	*Ambigolimax valentianus* (A. Férussac, 1821)	gastropods	1	100.0
Maria_Jimenez	28.504414, −16.230469	*Gallotia galloti* (Oudart, 1839)	lizards	20	10.0
Maria_Jimenez	28.504414, −16.230469	*Pseudosuccinea columella* (Say, 1817)	gastropods	6	50.0
Maria_Jimenez	28.504414, −16.230469	*Rattus rattus* (Linnaeus, 1758)	rats	10	0.0
Masca_ravine	28.296554, −16.849540	*Deroceras invadens* Reise, Hutchinson, Schunack & Schlitt, 2011	gastropods	2	0.0
Masca_ravine	28.296554, −16.849540	*Pseudosuccinea columella* (Say, 1817)	gastropods	16	0.0
Puerto_de_la_Cruz	28.410114, −16.534761	*Pseudosuccinea columella* (Say, 1817)	gastropods	7	0.0
Realejo_Alto	28.372002, −16.597173	*Hemicycla bidentalis* (Lamarck, 1822)	gastropods	26	7.7
Realejo_Alto	28.372002, −16.597173	*Insulivitrina lamarckii* (A. Férussac, 1821)	gastropods	14	100.0
San_Andres	28.513704, −16.202306	*Deroceras reticulatum* (O. F. Müller, 1774)	gastropods	5	0.0
San_Andres	28.513704, −16.202306	*Otala lactea* (O. F. Müller, 1774)	gastropods	1	0.0
San_Andres_Barranquetas	28.525729, −16.204946	*Deroceras invadens* Reise, Hutchinson, Schunack & Schlitt, 2011	gastropods	3	100.0
San_Andres_Barranquetas	28.525729, −16.204946	*Pseudosuccinea columella* (Say, 1817)	gastropods	7	0.0
San_Andres_Barranquetas	28.525729, −16.204946	*Rattus rattus* (Linnaeus, 1758)	rats	3	33.3
San_Miguel_de_Abona	28.097864, −16.620743	*Otala lactea* (O. F. Müller, 1774)	gastropods	12	50.0
Tamaimo	28.272673, −16.839540	*Pseudosuccinea columella* (Say, 1817)	gastropods	10	40.0
Tanque	28.359145, −16.789473	*Gallotia galloti* (Oudart, 1839)	lizards	5	40.0

Locality	Coordinates	Species	Group	No.	% Prevalence
Tanque	28.359145, −16.789473	*Rattus rattus* (Linnaeus, 1758)	rats	2	0.0
Tegueste	28.519349, −16.325843	*Physella acuta* (Draparnaud, 1805)	gastropods	14	50.0
Tegueste	28.519349, −16.325843	*Theba pisana* (O. F. Müller, 1774)	gastropods	20	0.0
Tegueste_Las_Canteras	28.515440,- 16.317220	*Rattus norvegicus* (J. Berkenhout, 1769)	rats	2	50.0
Tegueste_ravine	28.525650,- 16.337280	*Gallotia galloti* (Oudart, 1839)	lizards	11	18.2
Tegueste_ravine	28.525650, −16.337280	*Rattus rattus* (Linnaeus, 1758)	rats	12	50.0
Tegueste_ravine_entrance	28.526112, −16.339395	*Ambigolimax valentianus* (A. Férussac, 1821)	gastropods	2	100.0
Tegueste_ravine_entrance	28.526112, −16.339395	*Deroceras invadens* Reise, Hutchinson, Schunack & Schlitt, 2011	gastropods	5	80.0
Tegueste_ravine_entrance	28.526112, −16.339395	*Theba pisana* (O. F. Müller, 1774)	gastropods	2	0.0
Tejina	28.535918, −16.356946	*Gallotia galloti* (Oudart, 1839)	lizards	16	6.3
Tejina	28.535918, −16.356946	*Physella acuta* (Draparnaud, 1805)	gastropods	20	0.0
Tierra_del_Trigo	28.352948, −16.802981	*Ambigolimax valentianus* (A. Férussac, 1821)	gastropods	20	0.0
Tierra_del_Trigo	28.352948, −16.802981	*Deroceras reticulatum* (O. F. Müller, 1774)	gastropods	21	19.0
Tierra_del_Trigo	28.352948, −16.802981	*Insulivitrina lamarckii* (A. Férussac, 1821)	gastropods	20	30.0
Tierra_del_Trigo	28.352948, −16.802981	*Rattus rattus* (Linnaeus, 1758)	rats	11	9.1
Tierra_del_Trigo	28.352948, −16.802981	*Rumina decollata* (Linnaeus, 1758)	gastropods	20	5.0
Valle_de_San_Lorenzo	28.095308, −16.648007	*Gallotia galloti* (Oudart, 1839)	lizards	22	4.5
Valle_de_San_Lorenzo	28.095308, −16.648007	*Rattus norvegicus* (J. Berkenhout, 1769)	rats	2	0.0
Valle_de_San_Lorenzo	28.095308, −16.648007	*Rattus rattus* (Linnaeus, 1758)	rats	6	0.0
Valle_de_San_Lorenzo	28.095308, −16.648007	*Rumina decollata* (Linnaeus, 1758)	gastropods	11	0.0
Valle_Jimenez_La_Cuesta	28.481273, −16.275988	*Otala lactea* (O. F. Müller, 1774)	gastropods	2	0.0
Valle_Jimenez_La_Cuesta	28.481273, −16.275988	*Theba pisana* (O. F. Müller, 1774)	gastropods	14	0.0

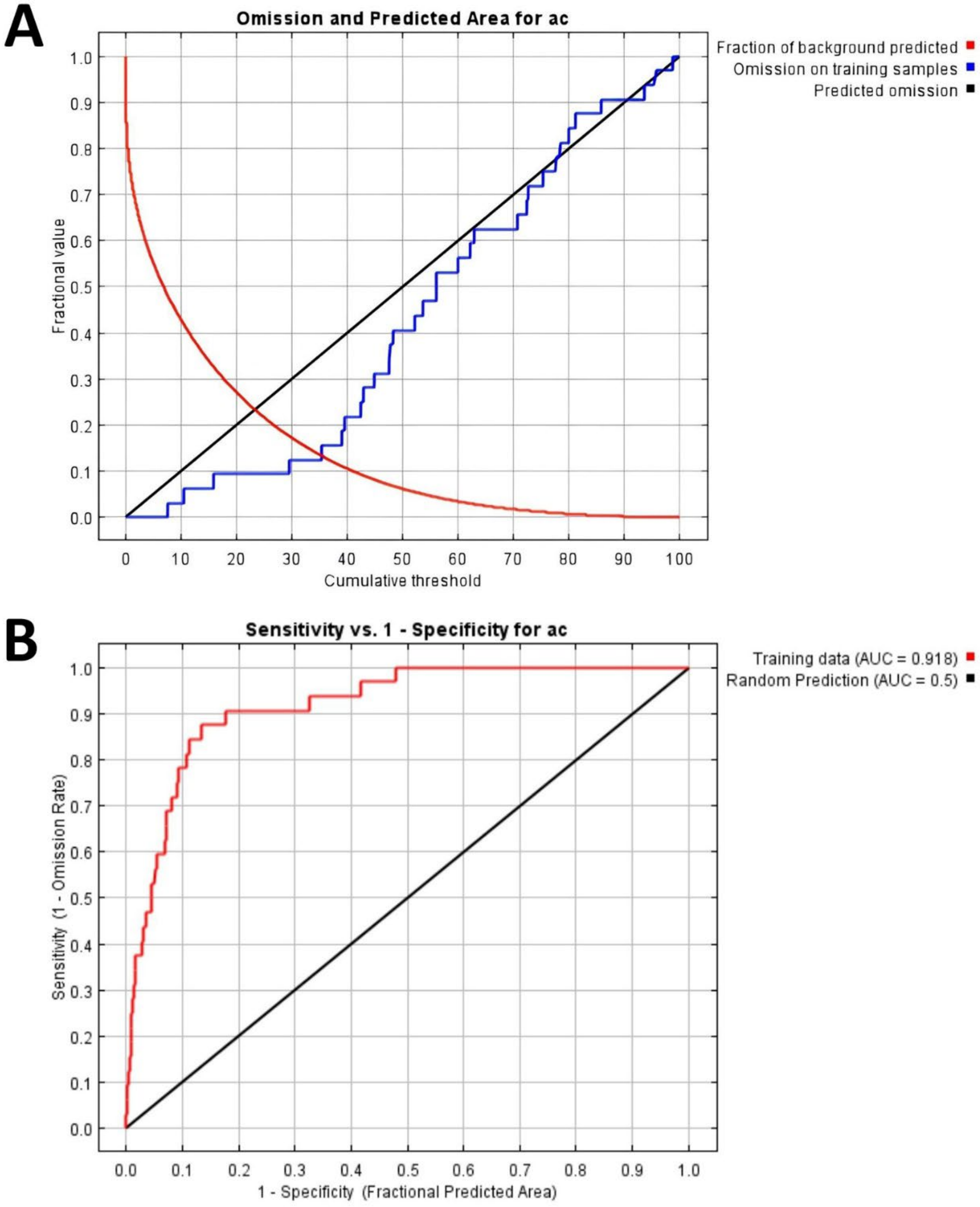

Appendix Figure 1. Model evaluation for MaxEnt. A) Evaluation of the MaxEnt model for *A. cantonensis*, based on occurrence data from Tenerife. Omission and predicted area curves showing the fraction of background predicted (red), omission on training samples (blue), and predicted omission (black) across cumulative thresholds. B) ROC curve of the MaxEnt model for *A. cantonensis* in Tenerife, showing model sensitivity versus 1 – specificity; the red line represents model performance (AUC = 0.918), while the black line represents random expectation (AUC = 0.5).

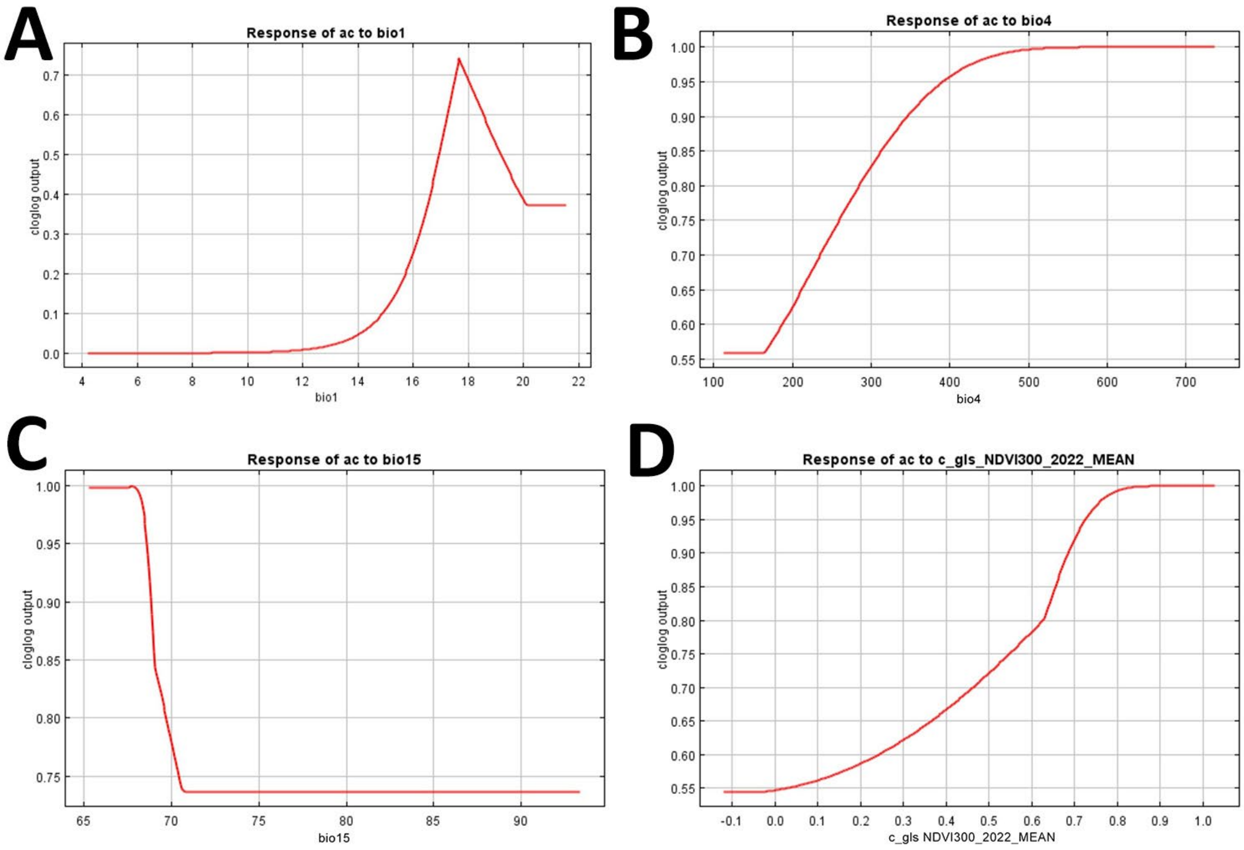

Appendix Figure 2. Response curves of the MaxEnt model for *A. cantonensis*, illustrating how key environmental predictors influence habitat suitability across Tenerife. A) bio1; B) bio4; C) bio15; D) NDVI mean. Each plot shows the predicted logistic output as a single environmental variable is varied while all others are held at their average sample value.

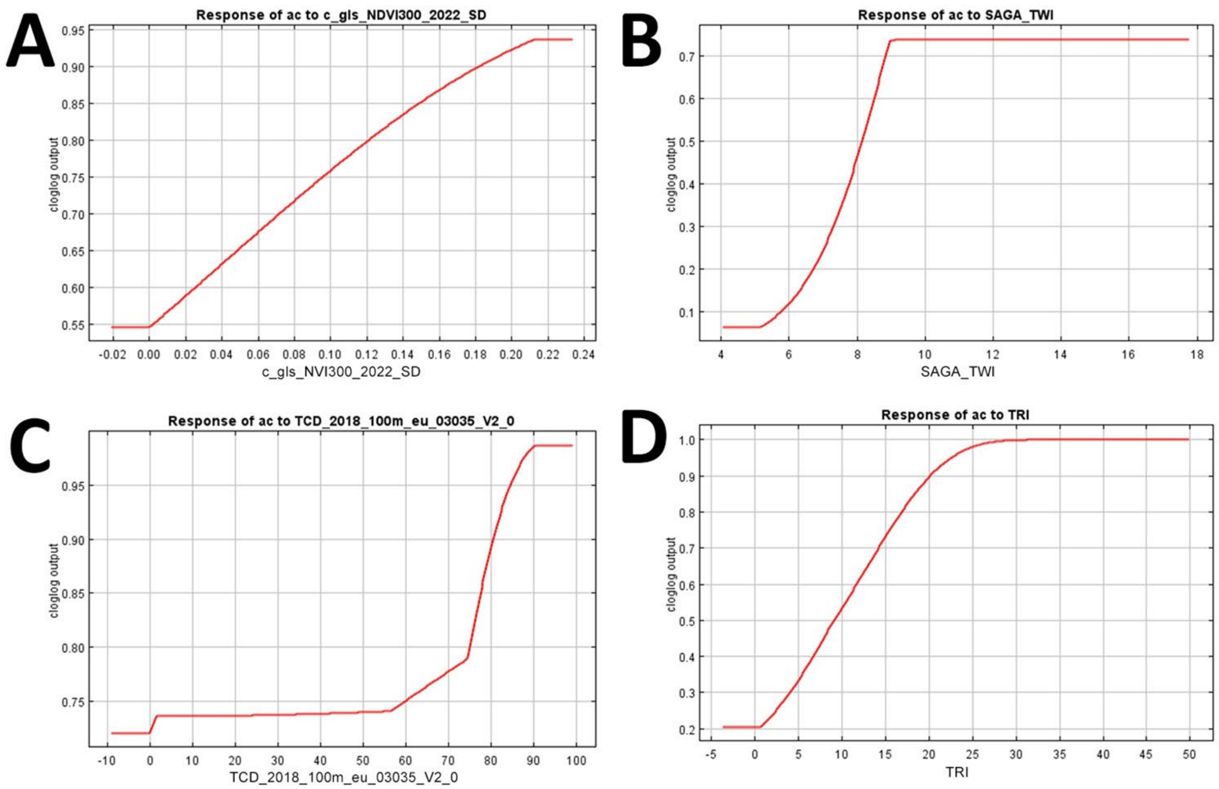

Appendix Figure 3. Response curves of the MaxEnt model for *A. cantonensis*, illustrating the effects of vegetation and topographic predictors on habitat suitability across Tenerife. A) NDVI standard deviation; B) topographic wetness index; C) terrain ruggedness index; D) tree cover density. Each plot shows the predicted logistic output as a single environmental variable is varied while all others are held at their average sample value.
